# Control of Tissue Growth and Cell Transformation by the Salvador/Warts/Hippo Pathway

**DOI:** 10.1371/journal.pone.0031994

**Published:** 2012-02-16

**Authors:** Xiaomeng Zhang, Felix A. Grusche, Kieran F. Harvey

**Affiliations:** 1 Cell Growth and Proliferation Laboratory, Peter MacCallum Cancer Centre, East Melbourne, Victoria, Australia; 2 Sir Peter MacCallum Department of Oncology, and Department of Pathology, University of Melbourne, Parkville, Victoria, Australia; University of Dayton, United States of America

## Abstract

The Salvador-Warts-Hippo (SWH) pathway is an important regulator of tissue growth that is frequently subverted in human cancer. The key oncoprotein of the SWH pathway is the transcriptional co-activator, Yes-associated protein (YAP). YAP promotes tissue growth and transformation of cultured cells by interacting with transcriptional regulatory proteins via its WW domains, or, in the case of the TEAD1-4 transcription factors, an N-terminal binding domain. YAP possesses a putative transactivation domain in its C-terminus that is necessary to stimulate transcription factors *in vitro*, but its requirement for YAP function has not been investigated in detail. Interestingly, whilst the WW domains and TEAD-binding domain are highly conserved in the *Drosophila melanogaster* YAP orthologue, Yorkie, the majority of the C-terminal region of YAP is not present in Yorkie. To investigate this apparent conundrum, we assessed the functional roles of the YAP and Yorkie C-termini. We found that these regions were not required for Yorkie's ability to drive tissue growth *in vivo*, or YAP's ability to promote anchorage-independent growth or resistance to contact inhibition. However, the YAP transactivation domain was required for YAP's ability to induce cell migration and invasion. Moreover, a role for the YAP transactivation domain in cell transformation was uncovered when the YAP WW domains were mutated together with the transactivation domain. This shows that YAP can promote cell transformation in a flexible manner, presumably by contacting transcriptional regulatory proteins either via its WW domains or its transactivation domain.

## Introduction

The Yes-associated protein (YAP) is a transcription co-activator that mediates the transcriptional output of the Salvador/Warts/Hippo (SWH) pathway, which is a tumour suppressor pathway that was first identified in *Drosophila melanogaster* [1]. The SWH pathway restricts organ size in *D. melanogaster* and mammals, and deregulation of the pathway leads to egregious organ overgrowth [2], [3], [4]. In *D. melanogaster*, core components of the SWH pathway include the scaffold proteins, Salvador (Sav) and Mob as tumor suppressor (Mats), and the S/T kinases, Warts (Wts) and Hippo (Hpo). These proteins limit organ growth by phosphorylation-mediated inhibition of Yorkie (Yki), which is homologous to YAP in mammals [5]. In mammals, LATS1 and LATS2 (the homologs of *D. melanogaster* Wts) phosphorylate YAP on five sites, of which S127 and S381 appear to be the most important [6], [7]. S127 phosphorylated YAP partitions more readily to the cytoplasm through binding with 14-3-3 proteins [6], [7], while S381 phosphorylation leads to YAP destabilization through ubiquitin-mediated degradation [8]. Upstream of the core kinase cassette, an increasing number of proteins, many of which reside at cell junctions, have been shown to regulate SWH pathway activity [9].

Following the discovery that Yki promotes the growth of *D. melanogaster* tissues, several points of evidence have shown that YAP has oncogenic potential in mammals. Overexpression of YAP can confer anchorage-independent growth of NIH3T3 or MCF10A cells and can stimulate growth-factor independent growth, migration and invasion of MCF10A cells, which are hallmark properties of oncogenes [10], [11], [12]. In transgenic mice, YAP overexpression in liver, gastrointestinal tract and skin induces hyperplasia [6], [13], [14], whilst the *YAP* gene was found to be amplified in mouse models of breast and liver cancer [10], [15]. In addition, YAP protein is elevated and more nuclear at a high frequency in several types of human cancer, and increased nuclear YAP correlates with poor patient outcome in tumors such as ovarian, liver and lung [16], [17], [18], [19].

Although the mechanism of YAP-induced oncogenesis is not fully understood, several studies have suggested that the TEAD1-4 transcription factors are major mediators of YAP's growth-promoting ability. YAP activates TEAD1-4 and stimulates transcription of known TEAD1-4 target genes [20], [21]. In addition, gene-profiling studies showed a large degree of overlap of genes induced by overexpression of murine YAP or constitutively active TEAD2 [22]. The association between YAP and TEAD1-4 is mediated by the N-terminus of YAP and the C-termini of TEAD1-4 [21]. Reducing the expression of TEAD1-4, or destroying the interaction between YAP and TEAD1-4, blocks YAP-induced cell transformation [20]. Similarly, in *D. melanogaster*, the sole TEAD1-4 homologue, Scalloped (Sd), interacts with Yki and is required for Yki-induced tissue overgrowth [23], [24], [25]

As well as possessing a TEAD1-4-binding region in its N-terminus, YAP possesses several other conserved protein domains. It harbors two WW domains that mediate interactions with important regulatory proteins that either promote or inhibit YAP's ability to drive transcription and promote cell transformation, migration and epithelial to mesenchymal transition in a cell- and context-dependent fashion [11], [12], [26]. YAP also possesses binding motifs for SH3- and PDZ-domains, as well as a putative transactivation (TA) domain in its C-terminus [27], [28]. The TA domain was first identified based on its ability to transactivate the minimal Gal4 DNA-binding domain as well as the pEBP2α transcription factor [29], although its relationship to YAP's biological functions has never before been studied. Interestingly, homology between the C-termini of Yki and YAP is very low and they vary greatly in length; following the second WW domain, Yki contains a further 55 amino acids, whereas YAP contains 226–242 amino acids, depending on the splice variant. This low homology is in direct contrast to other regions of Yki and YAP, e.g. the WW domains, Sd/TEAD-binding domain and LATS/Wts phosphorylation sites, that are highly conserved [30], [31].

Therefore, we sought to investigate the requirement of the TA domain for YAP's ability to stimulate cell proliferation, migration, invasion and oncogenic transformation, as well as the C-terminus of Yki for its ability to stimulate tissue growth *in vivo*. Surprisingly, we found that the TA domain was dispensable for YAP-mediated transformation and proliferation. By contrast, this domain was required for YAP to stimulate cell migration and invasion. These results question an obligate requirement for the putative TA domain for YAP's ability to act as a transcriptional co-activator protein.

## Materials and Methods

### *Drosophila melanogaster* strains

**Figure 1:**
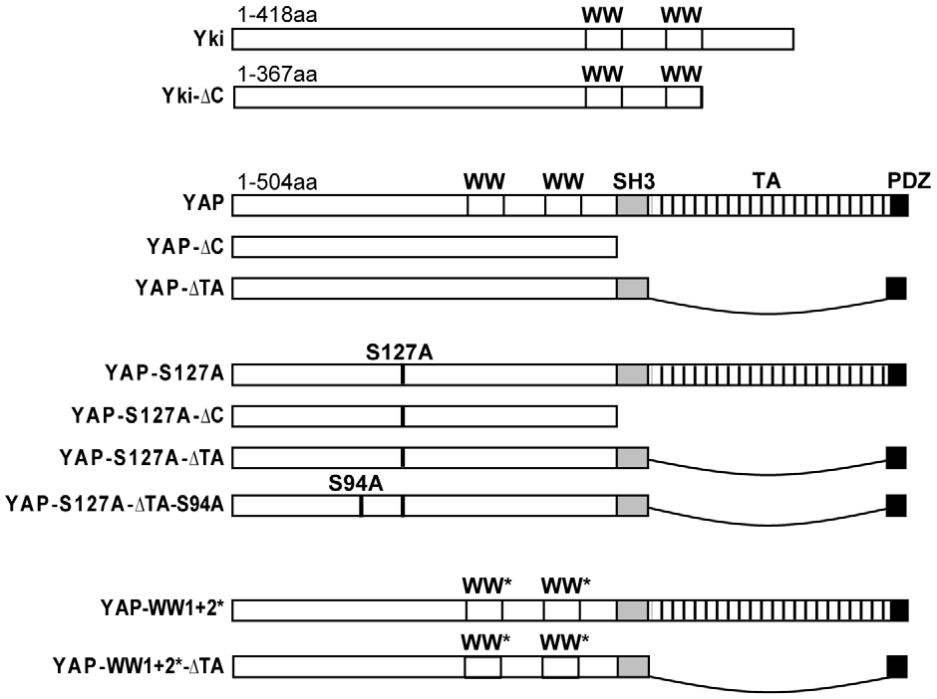
Schematic illustration of wild-type and mutant Yorkie and YAP proteins. Wild-type Yki is 418 amino acids long, whereas Yki-ΔC lacks the final 51 amino acids at the C-terminus. YAP2L is 504 amino acids long and contains two WW domains, as well as three domains in its C-terminus: an SH3 binding domain, a transactivation domain (TA) and a PDZ-binding motif. In YAP-ΔC, the C-terminus of YAP is deleted. In YAP-ΔTA, the TA domain is deleted. These deletions were generated in wild-type YAP2L, as well as in YAP2L-S127A, which contains a single amino acid mutation of S127 to A. In YAP-S127A-ΔTA-S94A, S94 is also mutated to A. YAP-WW1+2* includes W199F and P202A mutations in WW domain 1 and W258F and P261A mutations in WW domain 2. In YAP-WW1+2*-ΔTA, the WW domains are mutated as above and the TA domain is deleted.

**Figure 2:**
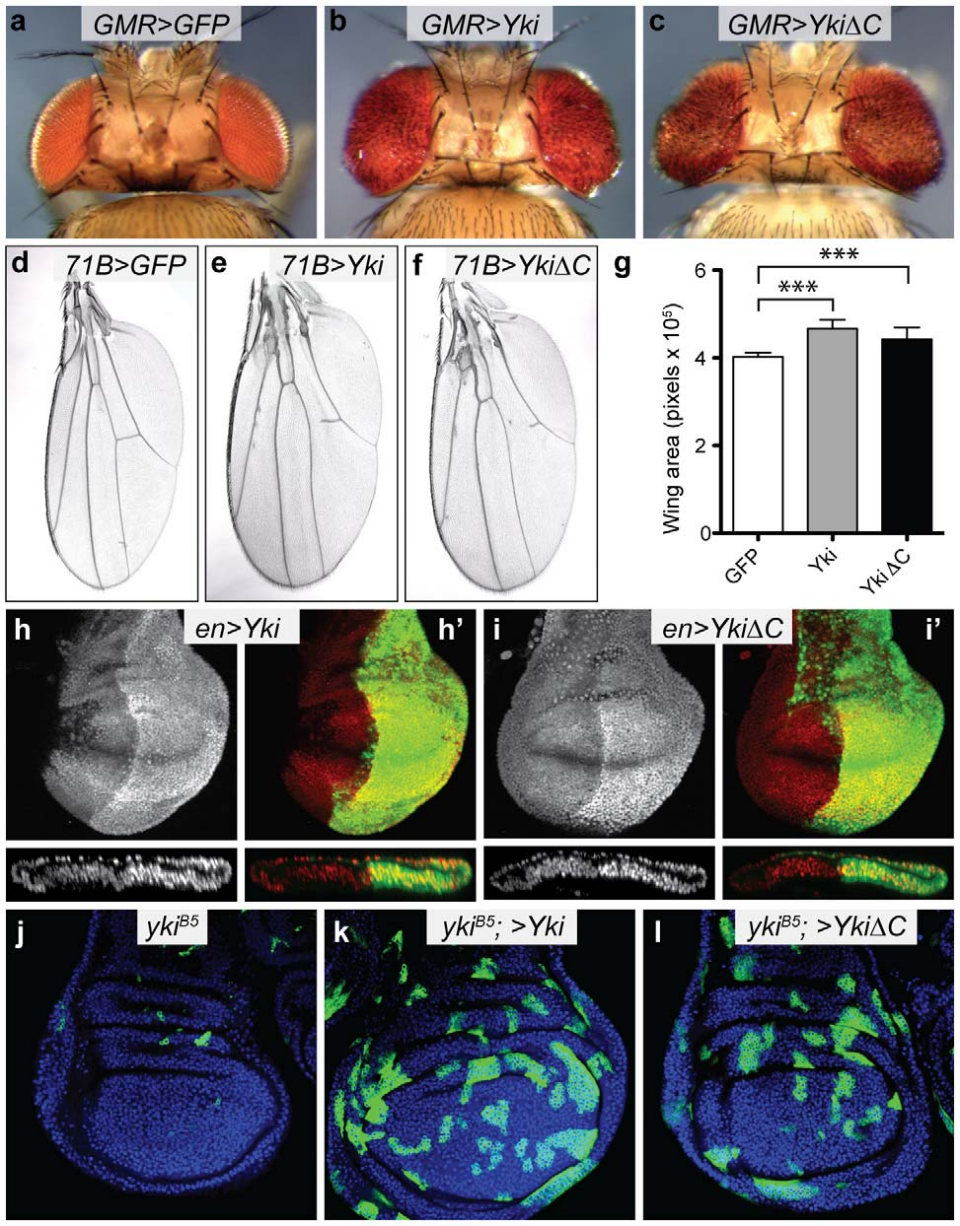
The carboxyl terminus of Yorkie is dispensable for its ability to stimulate tissue growth in *D. melanogaster*. (a–c) Dorsal views of fly heads expressing the indicated transgenes with the eye-specific *GMR-Gal4* driver. (d–f) Wings of flies expressing the indicated transgenes using the *71B-Gal4* driver. (g) Quantification of wing sizes of genotypes displayed in (d–f). Data is presented as mean +/− SD, n = 20 for each genotype, *** indicates p<0.0001. (h and i) Expression of *Yki* (h) and *Yki-ΔC* (i) in the posterior compartment of the developing wing (marked by GFP, green) with the *en-Gal4* driver resulted in upregulation of *ex-lacZ* (grayscale in single channel, red in overlay). (j–l) *yki^B5^* mutant clones alone or co-expressing a *yki* transgene in wing discs, marked by GFP (green). Nuclei of cells are marked with DAPI (blue). (j) *yki^B5^* mutant clones. (k) *yki^B5^* mutant clones co-expressing *Yki*. (l) *yki^B5^* mutant clones co-expressing *YkiΔC*.

Transgenic flies harbouring the *UAS-yki* or *UAS-ykiΔC* transgenes (represented schematically in Figure 1) were generated by phiC31-mediated targeted insertion into the VIE-260E site on chromosome 2L. Other were strains were: *GMR-Gal4, 71B-Gal4, ex^697^, yki^B5^* [5] *y w, hsFLP, UAS-CD8-GFP; tub-Gal4, FRT42D, tub-Gal80* [32]. *D. melanogaster* genotypes by Figure panel:

Figure 2a) *w; UAS-GFP/+; GMR-Gal4/+*

Figure 2b) *w; UAS-Yki/+; GMR-Gal4/+*

Figure 2c) *w; UAS- YkiΔC/+; GMR-Gal4/+*

Figure 2d) *w; UAS-GFP/+; 71B-Gal4/+*

Figure 2e) *w; UAS-Yki/+; 71B-Gal4/+*

Figure 2f) *w; UAS- YkiΔC/+; 71B-Gal4/+*

Figure 2h) *w; en-Gal4, UAS-GFP/UAS-Yki*

Figure 2i) *w; en-Gal4, UAS-GFP/UAS-YkiΔC*

Figure 2j) *w, hsFLP, UAS-CD8-GFP; tub-Gal4, FRT42D, tub-Gal80/FRT42D, yki^B5^*

Figure 2k) *w, hsFLP, UAS-CD8-GFP; tub-Gal4, FRT42D, tub-Gal80/UAS-Yki, FRT42D, yki^B5^*

Figure 2i) *w, hsFLP, UAS-CD8-GFP; tub-Gal4, FRT42D, tub-Gal80/UAS- YkiΔC, FRT42D, yki^B5^*

### Wing size measurements

Wings of male flies were mounted in Canada Balsam (Sigma) and imaged on an Olympus BX-51 microscope. Wing size was quantified using Adobe Photoshop CS3 as in [33]. Microsoft Excel was used for statistical analysis (two-sample students T-test assuming unequal variance).

### Immunohistochemistry

Imaginal wing discs were stained as described [34], using mouse anti-β-Galactosidase (1∶200; Sigma) and goat anti-mouse 555 (1∶600; Molecular Probes, Invitrogen). Images were recorded on an Olympus FV-1000 microscope and processed using Adobe Photoshop CS3. *yki^B5^* mutant clones (with and without *UAS-yki* transgene expression) were generated by a 15 min heat shock 72 hrs after egg deposition and were dissected 49 hrs later.

### Mammalian cell culture and analysis

MCF10A and NIH-3T3 cell transfection and infection were performed as described previously [11], [35]. Protein expression was determined by immunoblotting with anti-Flag (Sigma) and anti-Actin (Cell Signaling) antibodies. Two-dimensional culture of NIH-3T3 cells, three-dimensional culture of MCF10A cells, soft agar, cell proliferation, migration and invasion assays were performed using published protocols [11], [35].

### Expression constructs

YAP-S127A, YAP-WW1+2* and YAP-S94A mutations were described previously [11]. ΔC and ΔTA mutations were generated in either YAP2L or YAP2L-S127A by PCR amplification and cloned into pBabe. Yki-ΔC was generated by PCR and cloned into PKC26. All plasmids were verified by sequencing. Primer sequences were:

YAP-F: CCGGATCCACCATGGACTACAAAGACGATGACGACAAGATGGACCCCGGGCAGCAG

YAP-ΔC-R: CCGAATTCCTAAGCACTCTGACTGATTCTC

YAP-ΔTA-R: CCGAATTCCTATAACCATGTAAGAAAGCTTTCTGGGCTCTGGGGAGCCAG

CCGAATTCCTATAACCATGTAAGAAAGCTTTCAGCACTCTGACTGATTCTC

Yki-Myc-F: CCGAATTCATGGAACAAAAACTCATCTCAGAAGAGGATCTGTGCGCGTGCCTAATC

Yki- ΔC-R: CCTCTAGATTATTGCATTCTGGGATCATTCC

## Results

### The carboxyl terminus of Yorkie is dispensable for its ability to stimulate tissue growth in *Drosophila melanogaster*

Homologues of YAP are present in many species from holozoans to humans [30], [31]. Among the most conserved domains in YAP homologues are the WW domains, the N-terminal TEAD-binding domain and regions of the C-terminus [30], [31], which have been posited to constitute YAP's transactivation domain [29]. Surprisingly, whilst *D. melanogaster* Yki displays strong conservation of the WW and TEAD-binding domains, it is bereft of the majority of C-terminal sequences found in YAP and has only 55 amino acids following the second WW domain compared with 242 amino acids in YAP2L. Despite this, it is still formally possible that the shorter Yki C-terminus possesses the ability to regulate transcription factor activity and tissue growth.

To investigate this possibility, we generated two *UAS*-inducible *yki* transgenes; one encoding a full length Yki protein and one encoding a Yki protein lacking the last 51 amino acids (Yki-ΔC) (Figure 1). Each transgene was inserted into the same genomic locus on chromosome II to ensure comparable overexpression. When overexpressed in the developing *D. melanogaster* eye using *GMR-Gal4*, each transgene caused strong overgrowth (Figures 2a–c). To verify these findings in another tissue, each transgene was overexpressed in the developing wing using *71B-Gal4*. Overexpression of *UAS-Yki* or *UAS-Yki-ΔC* increased wing size relative to the control by 16.2% and 9.9%, respectively, showing that each transgene caused tissue overgrowth in a different tissue type (Figures 2d–g). Yki drives expression of genes such as *DIAP1* and *expanded* (*ex*). To determine whether the C-terminus of Yki was required for Yki to regulate *ex* transcription, we assessed *ex* levels using a *lacZ* enhancer-trap in the *ex* locus in the posterior compartment of the wing. In the presence of either *yki* transgene, *lacZ* levels were elevated to similar degrees, showing that Yki does not require its C-terminus to regulate transcription from the *ex* locus (Figures 2h–i′). Finally, we investigated the ability of *yki-ΔC* to overcome growth and survival deficiencies associated with *yki* loss of function clones [5] by expressing each transgene in *yki^B5^* clones using the MARCM technique [32]. Both the *yki* and *yki-ΔC* transgenes rescued the size of *yki* clones to similar degrees (Figures 2j–l). Collectively, this data conclusively demonstrates that Yki does not require is C-terminus to promote tissue growth and survival, or transcription of a well-characterized Yki target gene.

### YAP does not require its transactivation domain to transform cells

The ability to induce anchorage-independent cell growth is a hallmark of oncogenes; this includes YAP, which has been shown to promote anchorage-independent growth of both MCF10A and NIH-3T3 cells [10], [11], [12], [36]. To investigate the functional role of the YAP C-terminus, we first sought to determine whether it is required for YAP to induce anchorage-independent growth. All of the following experiments were performed with *YAP2L* or derivatives of the *YAP2L* gene, but for simplicity, are referred to as *YAP*. Two mutants lacking either the entire C-terminal domain following the second WW domain, YAP-ΔC (deletion of residues 278 to 504 in YAP2L), or the region previously defined as the YAP transactivation domain [29], YAP-ΔTA (deletion of residues 291 to 497 in YAP2L), were constructed in either wild type YAP2L or in YAP2L-S127A, a hyperactive YAP where the major LATS1/LATS2 phosphorylation site has been mutated from S to A (Figure 1). Stably infected MCF10A or NIH-3T3 cells were generated that exhibited robust expression of wild type YAP or mutant YAP proteins (Figure S1).

**Figure 3:**
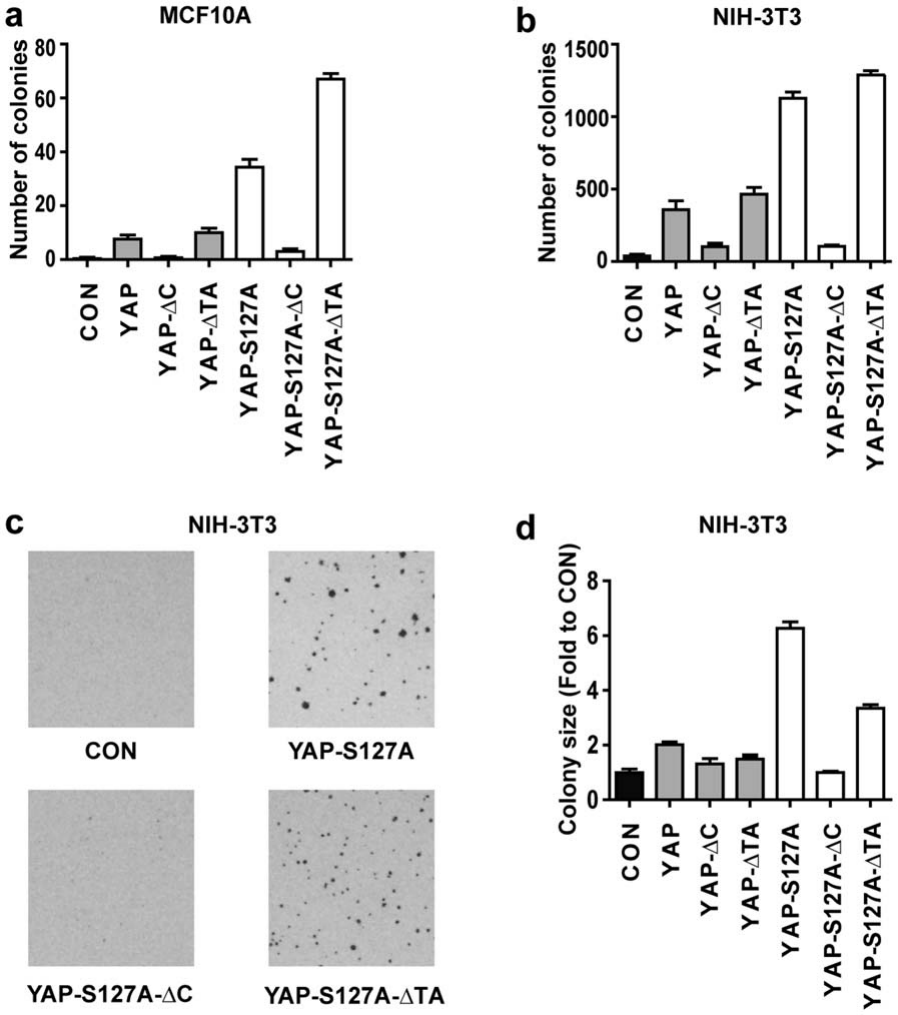
YAP does not require its transactivation domain to transform cells. (a and b) Quantitation of the number of colonies expressing vector alone (CON) or the indicated YAP plasmids in MCF10A (a) or NIH-3T3 (b) cells grown in soft agar. (c) Representative pictures of soft agar assays from (b). (d) Quantitation of the size of colonies expressing the indicated plasmids in NIH-3T3 cells grown in soft agar. Data in a, b and d are presented as mean +/− SD, n = 3.

To assess the ability of different YAP mutants to confer anchorage-independent cell growth, each cell line was plated in soft agar assay and incubated for 7 (NIH-3T3) or 14 (MCF10A) days. Consistent with previous reports, YAP induced robust colony growth in soft agar compared with the vector control. By contrast, YAP-ΔC failed to induce growth in soft agar (Figure 3a). Similarly, YAP-S127A-ΔC induced minimal colony growth in soft agar compared to YAP-S127A (Figure 3a). Unexpectedly, we observed no significant difference in colony number between cells expressing either YAP-ΔTA or YAP. In addition, YAP-S127A-ΔTA stimulated growth of a greater number of colonies in soft agar than YAP-S127A (Figure 3a). Similar results were obtained when the described YAP proteins were expressed in NIH-3T3 cells with respect to colony number in soft agar, however colony size was reduced in cells expressing YAP-S127A-ΔTA compared with YAP-S127A (Figures 3b–d). These results show that, unexpectedly, the YAP TA domain is not required for its ability to induce anchorage-independent growth and instead implicate the TA domain in mediating negative regulation of YAP activity.

### Transactivation domain-deficient YAP requires TEAD transcription factors to transform cells

**Figure 4:**
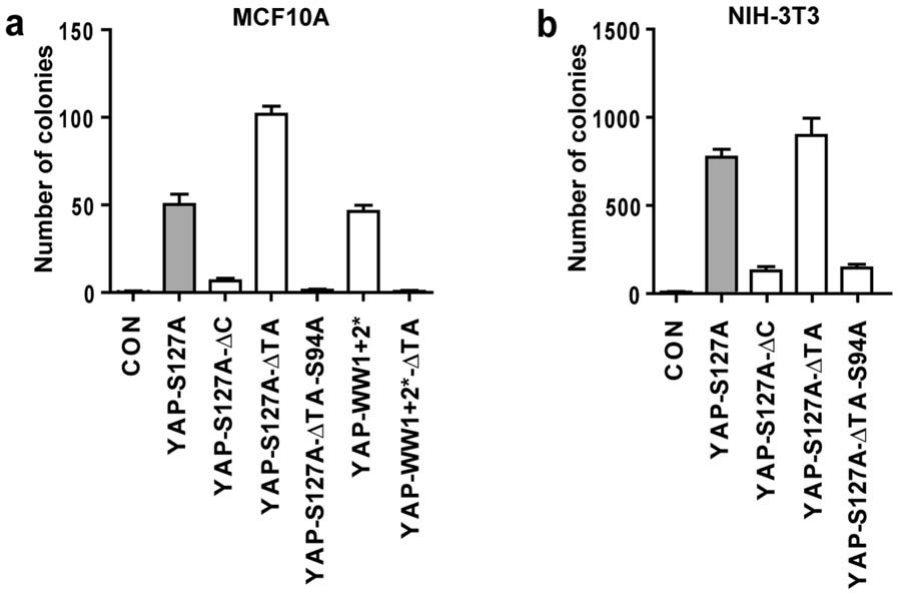
Hyperactivated YAP requires WW domains and TEAD transcription factors to stimulate cell transformation. Quantitation of number of colonies expressing vector alone (CON) or the indicated YAP plasmids in MCF10A cells (a) or NIH-3T3 cells (b). Data is presented as mean +/− SD, n = 3.

TEAD1-4 transcription factors are major effectors of the oncogenic activity of YAP [20]. Therefore, we sought to determine whether YAP-ΔTA and YAP-S127A-ΔTA-mediated cell transformation was reliant on TEAD transcription factors. Given that S94 of YAP mediates interaction with TEAD1-4 and mutation of S94 to A abolishes YAP's transformation potential [20], we investigated whether S94 was also required for YAP-ΔTA to transform cells. Stably transfected MCF10A and NIH-3T3 cells were generated that expressed YAP-S127A-ΔTA-S94A. When these cell lines were plated in soft agar, the S94A mutation was found to almost completely revert the ability of YAP-ΔTA to induce growth in soft agar of both MCF10A cells (Figure 4a) and NIH-3T3 cells (Figure 4b). These results show that, despite the deletion of the TA domain, YAP retains the ability to transform cells in a TEAD-dependent fashion.

### YAP-mediated cell transformation requires either the WW domains or its transactivation domain

Previously, we discovered that the WW domains of YAP and its *D. melanogaster* orthologue, Yki, played cell-specific roles with respect to induction of cell transformation and tissue growth, respectively [11]. Mutation of the YAP WW domains increased the ability of YAP to transform MCF10A cells, but reduced YAP's ability to transform NIH-3T3 cells. WW domain mutations also blocked Yki's ability to stimulate tissue growth [11]. These results suggested that the YAP/Yki WW domains interact with a protein(s) that is required for its ability to promote gene transcription in a cell-specific manner. Based on these earlier findings and our discovery that in MCF10A cells the TA domain of YAP is not required to induce growth in soft agar, we hypothesized that YAP activates transcription factors either by its TA domain or by proteins that interact with its WW domains. Therefore, we reasoned that mutation of both the TA domain and the WW domains would render YAP unable to transform MCF10A cells. Consistent with previous results, WW domain mutant YAP (YAP-WW1+2*) induced growth in soft agar with similar potency as YAP-S127A (Figure 4a) [11]. Deletion of the TA domain increased the ability of YAP-S127A to induce growth in soft agar even further. By contrast, when both the WW domains were mutated and the TA domain was deleted (YAP-WW1+2*-ΔTA), YAP's transforming potential was abolished (Figure 4a). This shows that in MCF10A cells, YAP can tolerate mutation of either the WW domains or the TA domain and in fact, each of these mutations alone causes YAP hyperactivation. However, YAP requires at least one of these domains to be intact to transform cells, which suggests inherent flexibility in YAP's ability to transform cells and activate TEAD transcription factors.

### The transactivation domain is not required for YAP to promote cell proliferation

**Figure 5:**
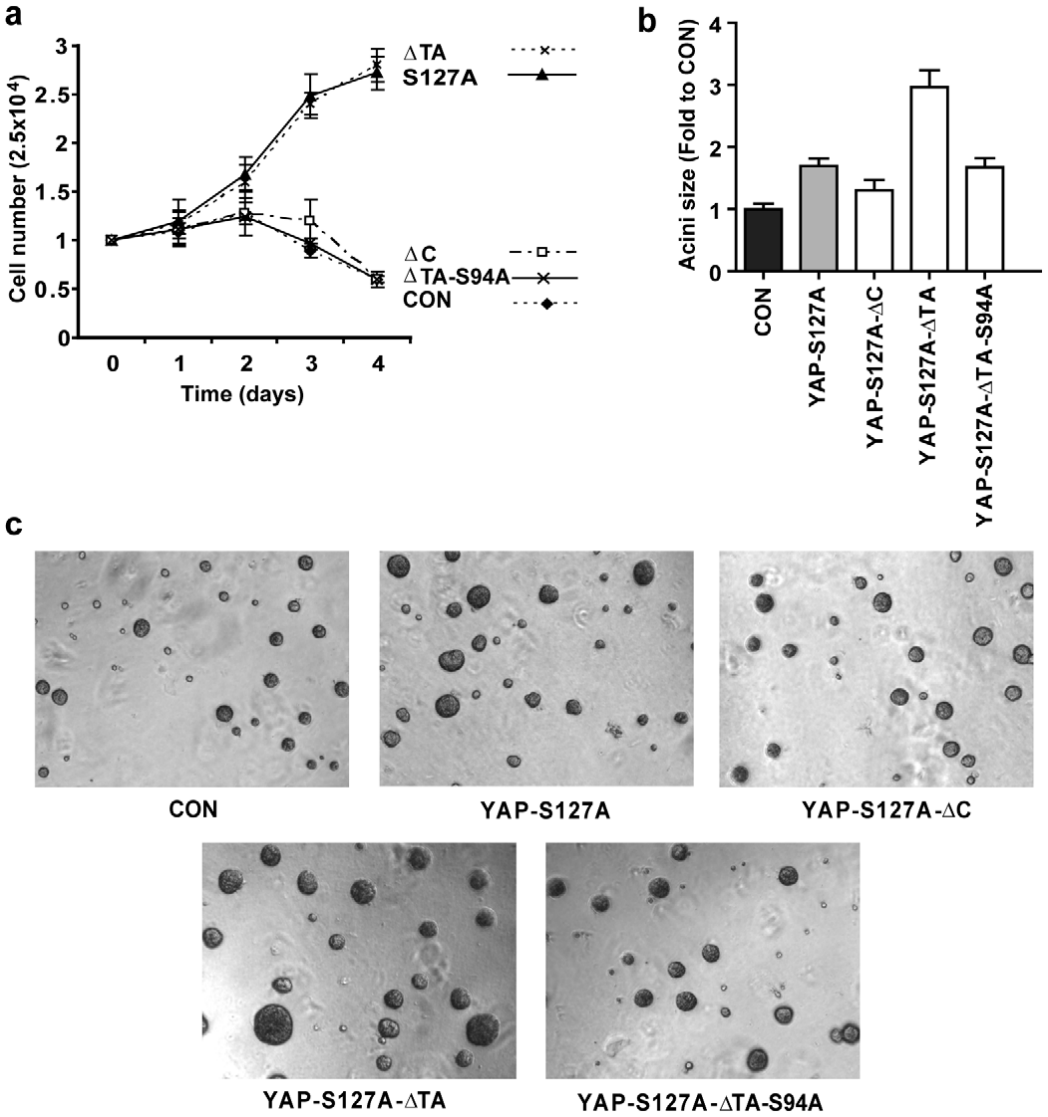
YAP's transactivation domain is dispensable for its ability to stimulate cell proliferation. (a) Proliferation rate of NIH-3T3 cells expressing vector alone (CON) or the indicated YAP plasmids when cultured in medium containing 0.5% serum. (b) Quantitation of the number of colonies expressing vector alone (CON) or the indicated YAP plasmids in MCF10A cells grown in soft agar for 14 days. Data in (a) and (b) is presented as mean +/− SD, n = 3. (c) Representative pictures of acini of MCF10A cells quantified in (b).

In previous studies, YAP was shown to enhance the proliferation rate of both NIH-3T3 cells grown in a two-dimensional (2D) culture, as well as MCF10A cell colony size grown in a three dimensional (3D) matrigel assay [11], [12], [22]. To investigate the role of different YAP protein domains for YAP's ability to increase proliferation rates, we assessed NIH-3T3 cells expressing different YAP variants plated in 0.5% serum. As shown in Figure 5a, the proliferation curve of stably infected NIH-3T3 cell lines could be classified into two groups: YAP-S127A and YAP-S127A-ΔTA both exhibited increased proliferation rates, whereas YAP-ΔC and YAP-S127A-ΔTA-S94A proliferated at a similar rate as vector control cells, and began to die after three days in 0.5% serum medium. This shows that deletion of the TA hyperactivates, rather than impedes, YAP's ability to increase rates of NIH-3T3 cell proliferation in low serum medium.

In addition, we assessed the size of acini formed by MCF10A cells that expressed different YAP variants, in 3D matrigel. As shown in Figures 5b and c, acini were larger when cells expressed YAP-S127A compared with vector control cells. However, additional deletion of the C-terminus reduced acini size of cells expressing YAP-S127A-ΔC, showing that the YAP C-terminus is required for this phenotype. Acini formed by cells expressing YAP-S127A-ΔTA were even larger than cells expressing YAP-S127A, further enforcing that the TA domain normally inhibits YAP function, consistent with our earlier results. In addition, the ability of YAP-S127A-ΔTA to stimulate MCF10A cell acini size was dependent on TEAD transcription factors, as introduction of the S94A mutation into YAP-S127A-ΔTA substantially reduced acini size (Figures 5b and c).

### The transactivation domain is essential for YAP to stimulate cell migration and invasion

**Figure 6:**
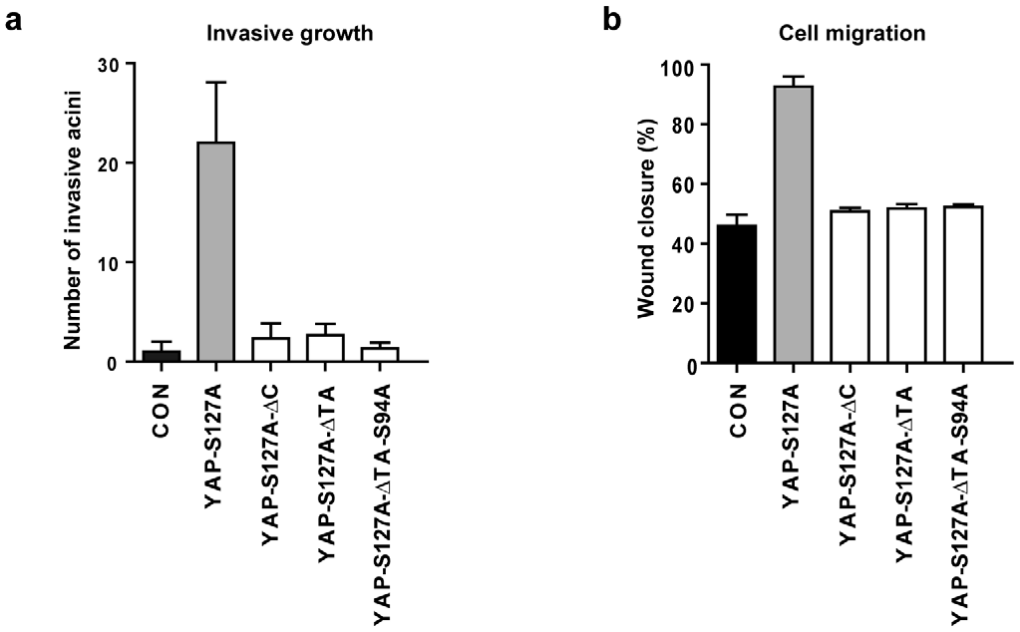
YAP requires its transactivation domain to promote cell migration and invasion. (a) Quantitation of acini with invasive protrusions in MCF10A cells expressing vector alone (CON) or the indicated YAP plasmids. (b) Quantitation of enclosed area after a scratch was introduced for 20 hours in confluent MCF10A cells expressing vector alone (CON) or the indicated YAP plasmids. Data are presented as mean +/− SD, n = 3.

To investigate the role of the TA domain of YAP in other functional settings, we assessed the ability of each YAP mutant to promote invasive growth in a 3D matrigel assay. Consistent with previous reports, overexpression of YAP-S127A in MCF10A cells cultured in matrigel caused spike-like protrusions, which is defined as an invasive phenotype [data not shown and [11]]. The number of invasive acini in matrigel was almost completely abolished in each of the YAP mutants (Figure 6a). We also assessed the ability of each YAP mutant to promote cell migration in a two-dimensional scratch assay. Cells were grown to confluence, scratched with a pipette tip and wound closure assessed 24 hours later. Cells expressing each mutant version of YAP migrated far less efficiently than cells expressing YAP-S127A, which exhibited enhanced migratory properties, consistent with our previous findings (Figure 6b) [11]. Taken together, these results show that the TA domain is required for YAP's ability to stimulate MCF10A cell invasion and migration, which contrasts with the requirement of this domain for anchorage-independent growth, colony growth and proliferation in low-serum media.

## Discussion

The Yorkie and YAP transcriptional co-activator proteins are conserved regulators of tissue growth in flies and mammals, respectively. Given the high degree of functional conservation between these proteins and the mechanism by which they are regulated, we were intrigued by the lack of conservation in the C-termini of these proteins, particularly because the C-terminus of YAP contains a transactivation domain that is necessary for its ability to activate transcription factors *in vitro* [29]. To investigate this apparent conundrum, we generated various YAP and Yki mutant proteins and assessed their activity using an array of functional assays.

Based on the previous finding that YAP possessed a potent TA domain that was required for YAP to activate the pEBP2α transcription factor [29], we predicted that it would be required for YAP to transform cells. Surprisingly, this was not the case as we found the TA domain to be dispensable for YAP's ability to induce transformation and proliferation of cells. In fact, this version of YAP displayed increased activity, pointing to the existence of YAP inhibitors that act via this domain. One such candidate is LATS1/2, which phosphorylates YAP S381 and primes it for ubiquitin-mediated degradation [8], although other inhibitors might also act via the TA domain.

By contrast, the TA domain was required for YAP to stimulate cell migration and invasive properties in cells cultured in 3D. This suggests that the YAP TA domain has context-specific roles: it is required for YAP to regulate genes that stimulate migratory behaviour, but not proliferation and survival. We previously observed a similar relationship when studying WW domain mutant versions of YAP in NIH-3T3 cells. A hyperactive version of YAP required its WW domains to stimulate cell migration, but not to promote growth in soft agar [11]. Alternatively, YAP might require different activity thresholds to regulate transcription of different classes of genes: e.g. a low threshold for genes that control cell survival and proliferation, and a high threshold for genes that control cell migration and invasion. The absence of the TA domain in Yki, might explain why, at least to date, Yki has not been found to regulate cell migration or motility in *D. melanogaster*.

Interestingly, when either the WW domains or TA domain were mutated, YAP's transformation potential increased substantially, but when both domains were mutated, YAP activity was lost. This suggests that YAP is a flexible transcription co-activator and that it can regulate transcription of genes that transform cells by complexing with proteins either through its WW domain or its TA domain. In either scenario, we found that YAP stimulates transformation via TEAD transcription factors. Our finding that the TA domain is not required to mediate transformation via TEAD transcription factors is supported by our study of Yki. The WW domains and the TEAD-binding domain are conserved between Yki and YAP, but the Yki and YAP C-termini are poorly conserved. The fact that a Yki protein that lacked the C-terminus could rescue loss of the wild-type protein shows that Yki activates its partner transcription factors independently of its C-terminus. Based on several recent studies, Yki most likely interacts with transcriptional regulatory proteins via its WW domains [37], [38]. This mechanism appears to have been conserved in mammalian YAP, with an additional layer of complexity, whereby YAP can promote transcription by interacting with proteins either via its WW domains or the TA domain.

The SWH pathway has been reported to be frequently subverted in human cancer, largely based on the observation that YAP is localized to the nucleus in a high percentage of solid tumours. Based on these findings and YAP's potent pro-tissue growth and pro-transformation activity, it has been touted as a candidate for therapeutic intervention. This study highlights a remarkable degree of complexity in both the regulation of YAP activity and the mechanism by which YAP regulates gene transcription. It also suggests redundant mechanisms by which YAP can regulate expression of genes that promote cell transformation, and that the same YAP domains mediate both positive and negative regulatory interactions. Therefore, great care will be required when designing therapies aimed at disabling YAP function in transformed cells.

## Supporting Information

Figure S1 **Expression of wild-type and mutant YAP proteins in MCF10A and NIH-3T3 cells.** Expression levels of YAP in cells stably expressing vector alone (CON) or various YAP proteins in either MCF10A or NIH-3T3 cells. Actin levels were determined to ensure even loading. Molecular mass markers in kDa are shown on the left.
(TIFF)
