# Supplementary figures and images for: Control of Tissue Growth and Cell Transformation by the Salvador/Warts/Hippo Pathway
Source: PLoS One. 2012 Feb 16;7(2):e31994. doi: 10.1371/journal.pone.0031994 (PMC3281119; doi:10.1371/journal.pone.0031994)

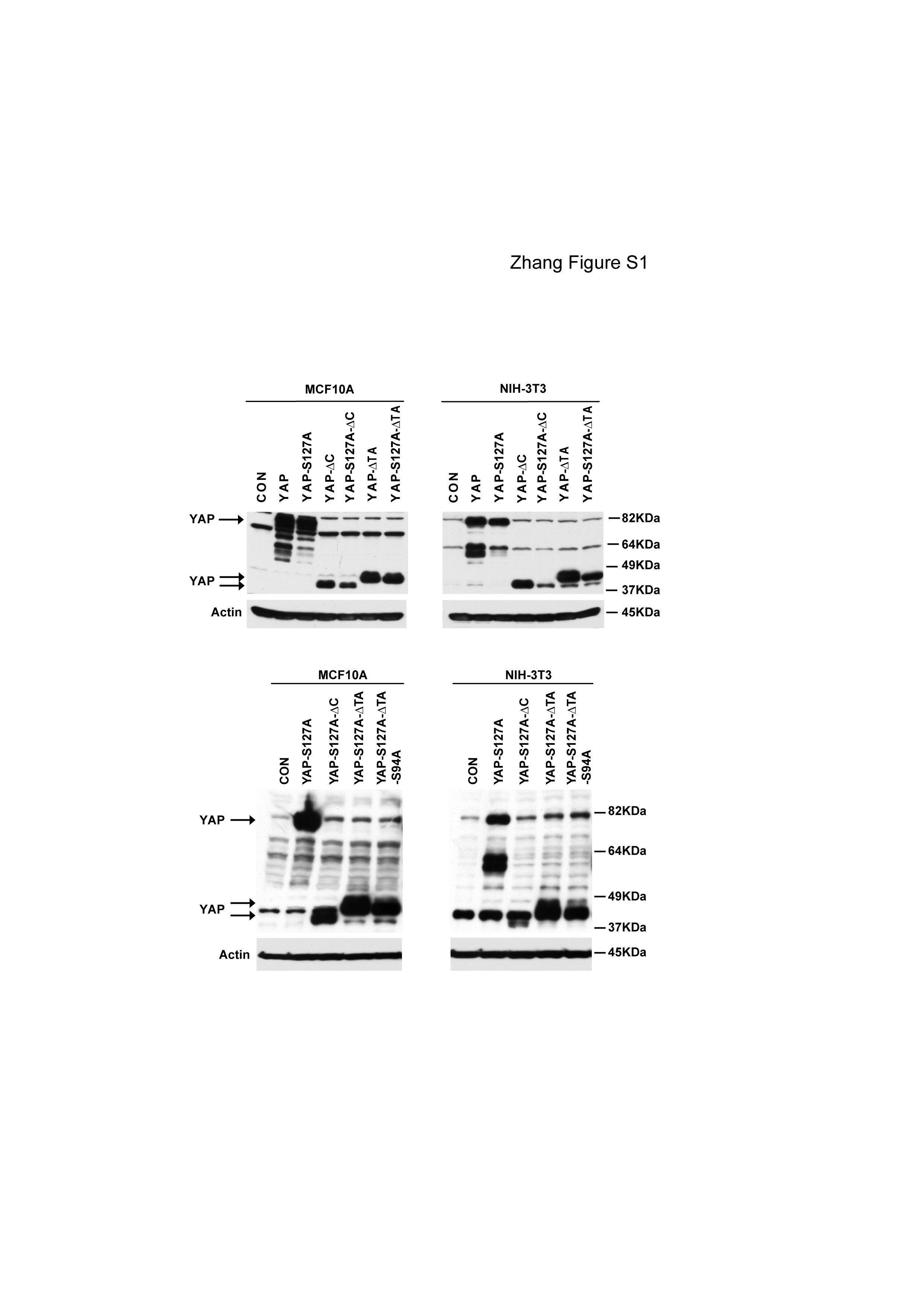

Supplement: Figure S1 — Expression of wild-type and mutant YAP proteins in MCF10A and NIH-3T3 cells. Expression levels of YAP in cells stably expressing vector alone (CON) or various YAP proteins in either MCF10A or NIH-3T3 cells. Actin levels were determined to ensure even loading. Molecular mass markers in kDa are shown on the left. (TIFF) [file pone.0031994.s001.tiff]
